# From habitat use to social behavior: natural history of a voiceless poison frog, *Dendrobates tinctorius*

**DOI:** 10.7717/peerj.7648

**Published:** 2019-09-17

**Authors:** Bibiana Rojas, Andrius Pašukonis

**Affiliations:** 1 Department of Biological and Environmental Science, University of Jyväskylä, Jyväskylä, Finland; 2 Department of Biology, Stanford University, Stanford, CA, USA; 3 Department of Cognitive Biology, University of Vienna, Vienna, Austria

**Keywords:** Agonistic behavior, Courtship, Parental care, Habitat use, Treefall, Tadpole transport, Amazon

## Abstract

Descriptive studies of natural history have always been a source of knowledge on which experimental work and scientific progress rely. Poison frogs are a well-studied group of small Neotropical frogs with diverse parental behaviors, distinct calls, and bright colors that warn predators about their toxicity; and a showcase of advances in fundamental biology through natural history observations. The dyeing poison frog, *Dendrobates tinctorius*, is emblematic of the Guianas region, widespread in the pet trade, and increasingly popular in research. This species shows several unusual behaviors, such as the lack of advertisement calls and the aggregation around tree-fall gaps, which remain poorly described and understood. Here, we summarize our observations from a natural population of *D. tinctorius* in French Guiana collected over various field trips between 2009 and 2017; our aim is to provide groundwork for future fundamental and applied research spanning parental care, animal dispersal, disease spread, habitat use in relation to color patterns, and intra-specific communication, to name a few. We report sex differences in habitat use and the striking invasion of tree-fall gaps; describe their courtship and aggressive behaviors; document egg development and tadpole transport; and discuss how the knowledge generated by this study could set the grounds for further research on the behavior, ecology, and conservation of this species.

## Introduction

Natural history has been long acknowledged as the foundation of new hypotheses in behavioral and evolutionary ecology (Endler, 2015). Thus, scientific progress relies greatly on knowing what different organisms are, where they live, what they feed on, how they respond to different stimuli, and what kind of other peculiar behaviors they exhibit (Tewksbury et al., 2014). Such knowledge requires data gathered through field observations of free-ranging animals.

Neotropical poison frogs (Dendrobatidae) and their close relatives are a showcase example of how detailed knowledge of natural history can lead to groundbreaking hypothesis-driven studies (e.g., Amézquita et al., 2011; Brown, Morales & Summers, 2010; Hegna et al., 2011; Pašukonis et al., 2014; Santos, Coloma & Cannatella, 2003; Saporito et al., 2007; Tarvin et al., 2017). Exhaustive field studies, in addition to detailed observations in captivity (Weygoldt, 1980, 1987; Zimmermann & Zimmermann, 1981, 1988) have revealed the diversity of poison frog parental care and social behaviors (e.g., Brust, 1993; Caldwell, 1996; Crump, 1972; Limerick, 1980; McVey et al., 1981; Summers, 1989; Wells, 1978, 1980), warning coloration (e.g., Myers & Daly, 1983; Silverstone, 1975), and skin alkaloids (e.g., Brodie & Tumbarello, 1978; Myers & Daly, 1976, 1980; Myers, Daly & Malkin, 1978), aspects that have become a trademark in the group both for research and for the pet trade. However, there is still a surprising lack of information on the natural history of some species that have become increasingly well studied otherwise, such as the dyeing poison frog, *Dendrobates tinctorius*.

Although bred in captivity by hobbyists for decades (Lötters et al., 2007; Schmidt & Henkel, 1995), and despite its growing status as a model species for studies on the evolution and function of coloration (e.g., Barnett et al., 2018; Lawrence et al., (in press); Noonan & Comeault, 2009; Rojas, Devillechabrolle & Endler, 2014; Rojas, Rautiala & Mappes, 2014; Wollenberg et al., 2008), there are only a handful of studies on *D. tinctorius* in its natural environment. Most of these have been carried out and published only after 2010 (Born et al., 2010; Courtois et al., 2012; Rojas, 2014, 2015; Rojas, Devillechabrolle & Endler, 2014; Rojas & Endler, 2013). Four other studies in the wild have attempted to understand evolutionary aspects of their variable coloration, using clay or wax models instead of the actual frogs (Barnett et al., 2018; Comeault & Noonan, 2011; Lawrence et al., (in press); Noonan & Comeault, 2009; Rojas, Rautiala & Mappes, 2014).

Many poison frog field studies over the last five decades have relied on prominent male calls either directly, by studying aspects related to vocal behavior (e.g., Erdtmann & Amézquita, 2009; Lüddecke, Fandiño & Amézquita, 1997; Forsman & Hagman, 2006; Lötters, Reichle & Jungfer, 2003; Vargas-Salinas & Amézquita, 2013), or indirectly, by using the calls to locate territorial males in the field (e.g., Amézquita et al., 2006; Bee, 2003; Hödl, Amézquita & Narins, 2004; Ringler et al., 2011; Rojas, Amézquita & Delgadillo, 2006). Meanwhile, *D. tinctorius* remained almost unstudied, at least in part, due to their lack of a regular calling behavior. Therefore, much of the behavioral and evolutionary ecology of dyeing poison frogs remains unknown.

As stated by the IUCN Red List for Threatened Species, *D. tinctorius* is in the category “Least Concern” (Gaucher & MacCulloch, 2010). According to this report, its major threat is illegal trading, as it is for various other dendrobatid species (Brown et al., 2011; Gorzula, 1996; Hoogmoed & Avila-Pirés, 2012; Nijman & Shepherd, 2010). In fact, because of its prominence in the pet trade, this and most species of poison frogs have long been listed in the Appendix II of CITES (CITES, 2017). However, a recent study provided evidence that, while their populations are seemingly large and stable throughout its range, *D. tinctorius* is not safe from the chytrid fungus (*Bd*) infection (which, incidentally, was discovered in a captive individual of *D. tinctorius*; Longcore, Pessier & Nichols, 1999) in its natural habitat (Courtois et al., 2012). Moreover, a recent study by Courtois et al. (2015) raised even greater concern as, of all the species tested for *Bd* in French Guiana, the highest prevalence was found in dendrobatid frogs, including *D. tinctorius*.

Alarming declines make it even more urgent to study the natural history of amphibian species and communities, especially of “sentinel” species such as *D. tinctorius* (Courtois et al., 2015), whose declines provide anticipated warning of risks to human or ecosystem health (Beeby, 2001). Only by understanding organisms in their own habitat can we produce sensible and timely conservation policies, and sustainable management (Caro, 1999; Tewksbury et al., 2014). In the particular case of *D. tinctorius*, the latest IUCN report of threatened species available indicates that research is needed on their population size, distribution and trends, as well as on their life history and ecology (Gaucher & MacCulloch, 2010). Thus, knowing their habitat use, breeding biology, social behavior, and movement ecology could be of utmost importance for modeling disease spread and the impacts of deforestation, among other current environmental threats. Here, we (1) document the habitat use, and the reproductive, social, and vocal behaviors of *D. tinctorius* in the wild; and (2) provide information about various other aspects of its natural history that will be a valuable groundwork for future fundamental and applied research in behavior, ecology, evolution, and conservation.

## Materials and Methods

### Study species

**Figure 1:**
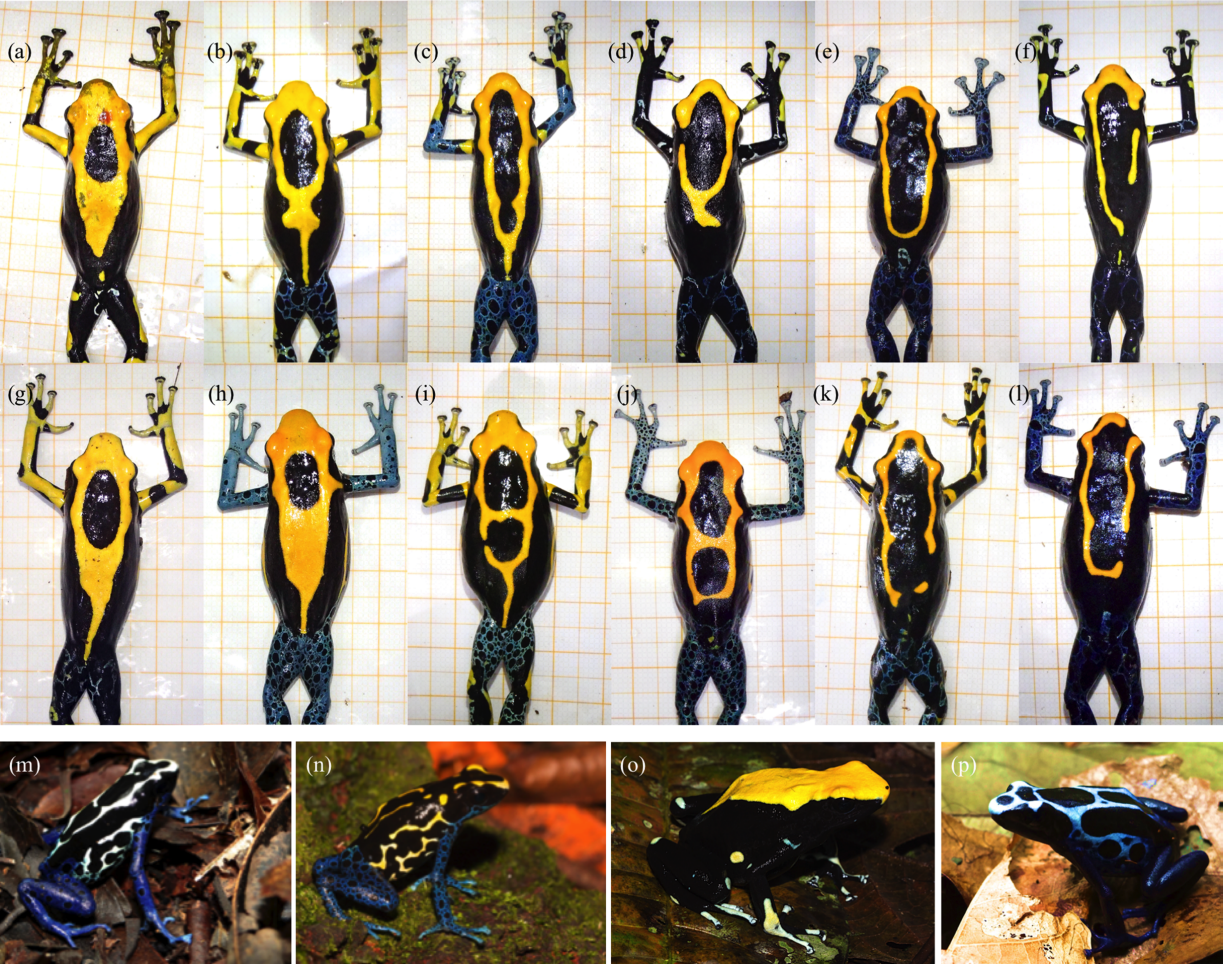
Colour pattern variation (A–L) within the studied population (as described in Rojas & Endler, 2013) and (M–P) between different populations of the dyeing poison frog in French Guiana (see Noonan & Gaucher, 2006). (A–F) males, (G–L) females. Lines on the background paper mark five mm. Note the enlarged toe discs in males, but overall larger female body size (for details see Rojas & Endler, 2013). Photo credits: Andrius Pašukonis and Matthias-Claudio Loretto ((A–L) Nouragues Nature Reserve, French Guiana), Antoine Fouquet ((N) Bakhuis, Suriname; (O) Mt. Galbao, French Guiana), and B. Rojas ((M) Mt. Matoury, French Guiana; (P) Mt. Bruyére, French Guiana).

*Dendrobates tinctorius* is a diurnal, relatively large (Snout-Vent Length 37—53 mm at the study site; Rojas & Endler, 2013) poison frog of the Neotropical family Dendrobatidae (more specifically, of the “*tinctorius* group”; Grant et al., 2006), which occurs around canopy gaps in primary forests in the Eastern Guiana Shield, at elevations between 0 and 600 m (Noonan & Gaucher, 2006; Wollenberg et al., 2006). It has skin alkaloids (Daly, Myers & Whittaker, 1987), and is characterized by a great color pattern variation both within (Figs. 1A–1I; Rojas & Endler, 2013) and among populations (Figs. 1M–1P; Noonan & Gaucher, 2006; Wollenberg et al., 2008).

**Figure 2:**
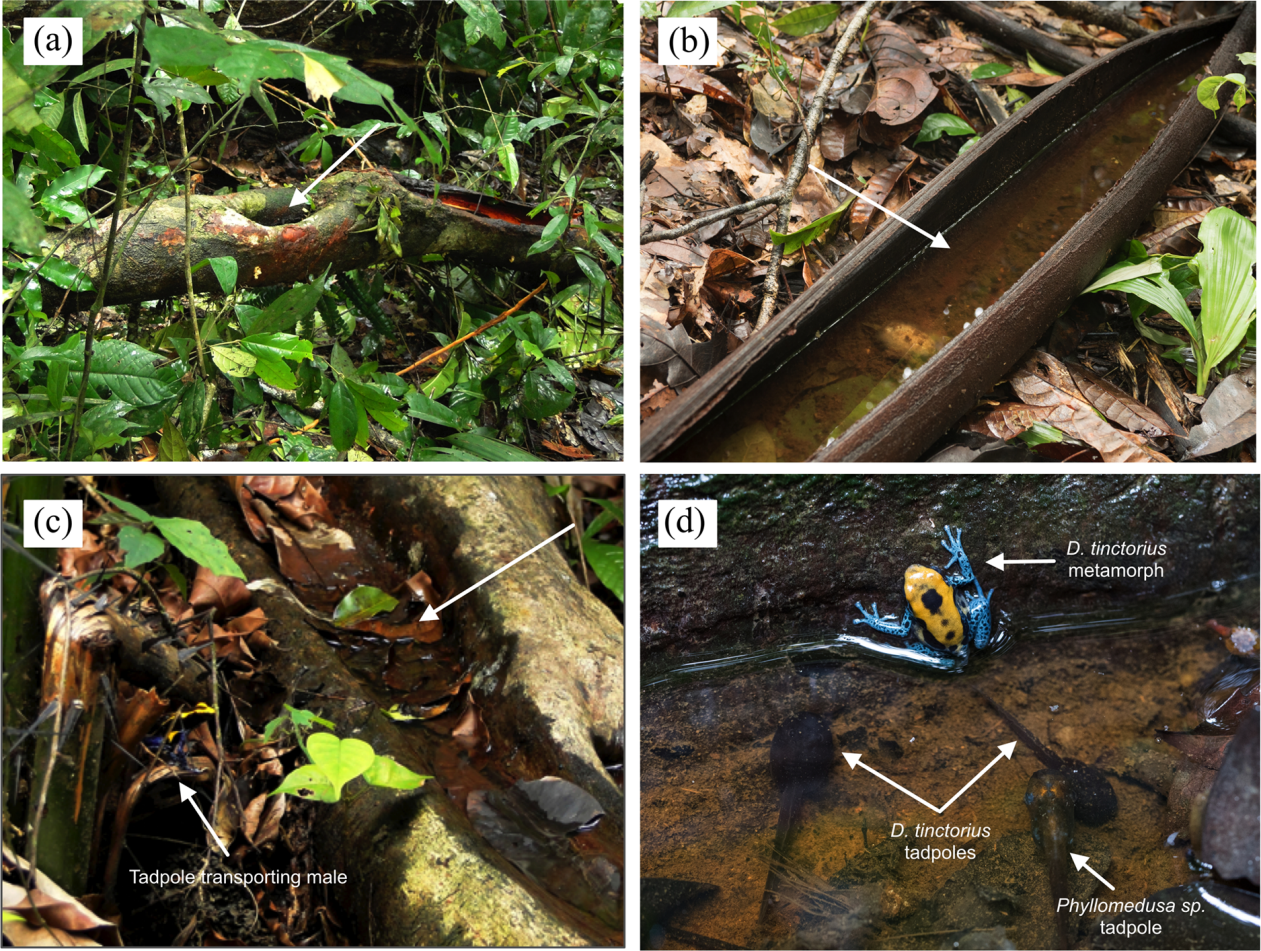
Examples of phytotelmata (pointed at by the arrows with no label) used as tadpole-deposition sites at the studied population. Some of these become available when a tree falls (A, C, D). Tadpoles of other species can sometimes be found sharing these pools with *D. tinctorius* tadpoles (D). Color patterns are clearly visible already at metamorphosis (D). Photo credits: Bibiana Rojas (A, C) and Andrius Pašukonis (B, D).

In our study area, color patterns can be used reliably for individual identification (Born et al., 2010; Courtois et al., 2012; Rojas & Endler, 2013; Figs. 1A–1l), and sex can be determined by the size of males’ toe discs, which are wider than females’ in relation to their body size (Rojas & Endler, 2013). In contrast to most frogs (including closely related poison frogs), male *D. tinctorius* do not produce advertisement calls, and when they do vocalize, they do it very softly (Lescure & Marty, 2000). Newly hatched tadpoles are carried by males to pools formed in tree holes or palm bracts at variable heights (Fig. 2; Video S1; Rojas, 2014, 2015), where they remain unattended until metamorphosis, which occurs after approximately two months (B. Rojas, 2011, personal observation in the field). As in some other species of *Dendrobates* (Caldwell & de Araújo, 1998; Gray, Summers & Ibáñez, 2009; Summers, 1990; Summers & McKeon, 2004), larvae feed on detritus and on larvae of insects and frogs (Rojas, 2014), including conspecifics (Rojas, 2014, 2015; Video S1). In captivity, individuals take up to 18 months to reach maturity (Lötters et al., 2007), but their age at sexual maturity in the field is unknown to date.

### Reproductive and social behavior

All the observations reported here were done at Camp Pararé, Nouragues Ecological Research Station, French Guiana (4°02′N, 52°41′W), in primary lowland terra-firme forest, where *D. tinctorius* is one of the most common leaf-litter frogs (Courtois et al., 2013). The diurnal frog community includes five other species of dendrobatid frogs (*Allobates femoralis, A. granti, Ameerega hahneli, Anomaloglossus baeobatrachus*, and *Ranitomeya amazonica*) and three bufonid species (*Atelopus aff. flavescences, R. castaneotica, R. lescurei*) (Born & Gaucher, 2001). Some of these species breed in, or take their tadpoles to, the same bodies of water used by *D. tinctorius* for tadpole deposition (Born & Gaucher, 2001; Rojas, 2014). In addition, several nocturnal hylids (*Trachycephalus resinifictrix*, *T. hadroceps*, *Osteocephalus oophagus*, *Phyllomedusa spp*., Gaucher, 2002; A. Pašukonis, 2016, 2017, 2019, personal observation; B. Rojas, 2010, 2011, personal observation) share some of the same breeding pools with *D. tinctorius*.

B. Rojas did systematic observations during three field seasons between January 9 and February 20 2009, January 17 and March 19, 2010, and January 17 and June 6 2011. In addition, A. Pašukonis made opportunistic observations on social and reproductive behavior at the same study site between January and March 2016 and 2017. The study periods correspond to the early rainy season and high reproductive activity of *D. tinctorius* in the study area.

During each study period between 2009 and 2011, B. Rojas surveyed a 1.5 km transect on a near-daily basis, between 8:00 and 17:30. Each frog found was captured, when possible, and photographed for future individual identification on the basis of its color patterns. When two individuals seemed to be interacting, they were followed for as long as it was necessary to determine the nature of the interaction (i.e., courtship or agonistic encounter). Two individuals were considered to be in courtship when they were less than one meter apart (as in Pröhl (2002)) and one was clearly following and touching the other (B. Rojas, 2009, 2010, 2011, personal observations) for at least 15 min. A 15-min waiting time was chosen on the basis of previous studies of mate choice and assortative mating in captive dendrobatids (Maan & Cummings, 2008, 2009). When possible, we followed pairs in courtship until they were no longer visible or until oviposition occurred, which proved difficult most of the time because of poor accessibility and visibility under forest structures. Agonistic encounters were more difficult to follow than courtship interactions because of their usually short duration and the high movement speed of the frogs, but we observed them for as long as both individuals were visible. Fragments of the two types of interactions were filmed for documentation purposes. Observations were done at irregular time intervals during the day.

Males carrying tadpoles were found during daily surveys along a 1.5 km transect. B. Rojas recorded the number of tadpoles on the back of each tadpole-carrying male and captured it when possible. Upon capture, each male was photographed (with the tadpole(s) still attached) against graph paper. Later these photos were used to measure the size of both the frog and the tadpoles with the software ImageJ (Schneider, Rasband & Eliceiri, 2012). Tadpole size was measured dorsally, from the tip of the snout to the base of the tail.

### Vocal behavior

*Dendrobates tinctorius* vocalizes rarely and at very low intensities, making it difficult to obtain audio recordings. We were able to obtain a high-quality audio recording of one male. In addition, to measure the acoustic properties of the call, we extracted lower quality audio from video recordings of social interactions. In total, we obtained sufficient quality recordings of eight calls produced by three males (four, three, and one call per individual). We manually measured the duration, pulse rate, and dominant frequency of each call using Praat (v. 5.3.85; Boersma & Weenink, 2014) acoustic analysis software. We averaged the measures between calls within each male and then between the three males. We used one call of the highest quality to visually illustrate call structure.

### Treefall-gap invasion

In a previous study, Born et al. (2010) reported frequent sightings of adult *D. tinctorius* in recently formed tree-fall gaps, but provided no quantitative information on the phenomenon. B. Rojas witnessed the formation of nine tree-fall gaps over the study periods (one in 2009, eight in 2011); these were discovered rapidly because they occurred in the 1.5 km transect surveyed daily. B. Rojas inspected each gap within the first 24 h of its formation and caught as many frogs as possible, moving fallen branches until no frogs were seen (after 2–3 h). During the next two consecutive days B. Rojas carefully searched for new frogs over a similar period of time (2–3 h). When frogs were seen but not caught, B. Rojas photographed them from a distance to record their color pattern for further identification upon capture. Two days after treefall occurrence, one, two, or three bowls with water were added at six of the newly formed gaps (depending on the gap’s size) during the course of a parallel study (Rojas, 2015). These bowls were meant to simulate newly available tadpole-deposition sites.

### Habitat use

During the field season of 2010, B. Rojas captured 109 frogs (55 females and 54 males), each of which was assigned to one of two microhabitats according to where they were first seen: leaf litter (when frogs were on a relatively open patch of leaf litter without any obvious structure in a one m radius), or associated to the following structures: fallen logs (when frogs were visibly exposed on top of the log or inside hollow trunks), fallen branches (when individuals were in fallen tree crowns), and tree roots (when the frogs were within the exposed roots or next to them). Frogs were only included in the analyses once (recaptures of the same individual were excluded in order to avoid pseudoreplication, and only the site at first sighting was taken into account). We tested for differences between the sexes in the microhabitat where they were found (open vs. associated with the aforementioned structures) using a Generalized Linear Model with binomial distribution. All statistical analyses were done with the software R v. 3.3.3 (R Core Team, 2013) using the RStudio interface (RStudio Team, 2015).

### Ethics statement

Our research was approved and authorized by the scientific committee of the Nouragues Ecological Research Station. We strictly adhered to the current French and European Union law, and followed the Association for the Study of Animal Behaviour’s (ASAB) Guidelines for the use of live animals in teaching and research (ASAB, 2017).

## Results

During three field seasons between 2009 and 2011, we identified 629 individuals unequivocally, 597 of which were captured. We photographed the remaining 32 frogs from a distance that allowed the record of their unique color patterns and, thus, their individual identification. There was no statistically significant difference between the number of females (*N* = 276) and the number of males (*n* = 321) found, although there was a non-significant trend towards a larger number of males (χ^*2*^= 3.392, df = 1, *P* = 0.066).

### Habitat use

**Figure 3:**
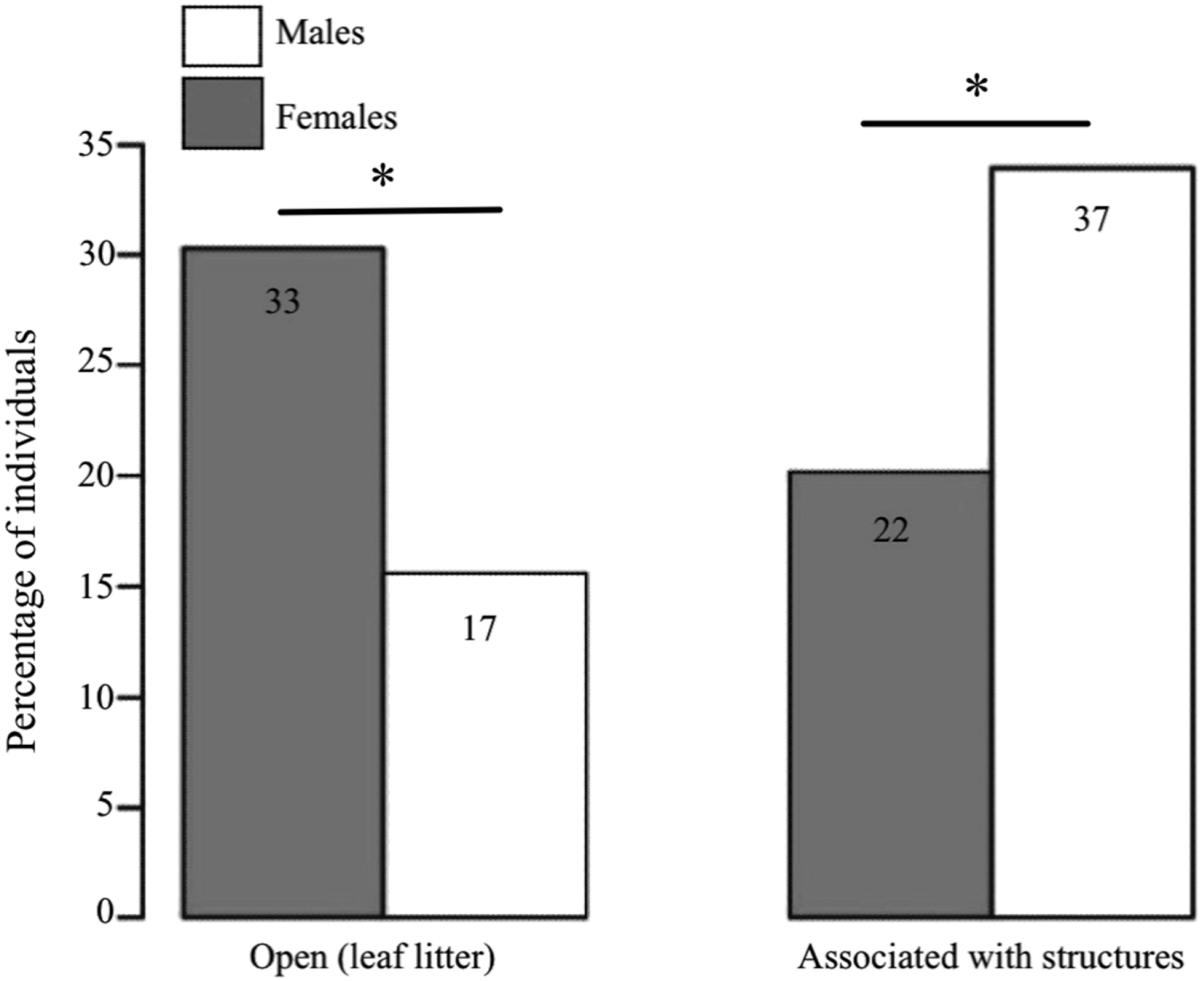
Habitat use in *D. tinctorius* in relation to sex. Numbers in the boxes indicate the total number of individuals in each category (*N* =109). Females are more often associated with open areas of leaf litter, whereas males are more frequently found associated to structures such as fallen logs and buttresses (*Z* = 2.943, *P* < 0.001). Asterisks denote significant differences at the 0.05 level. Data collected only in 2010.

We found clear differences between the sexes in terms of the microhabitat where they were found (GLM: estimate ± SE = 1.183 ± 0.402, *Z* = 2.943, *P* < 0.001, *n* = 109; Fig. 3). Females were predominantly found in open areas of leaf litter (60% of females vs. 31.5% of males), whereas males were mostly found associated to structures (68.5% of males vs. 40% of females), such as fallen logs and branches.

### Invasion of treefall gaps

**Figure 4:**
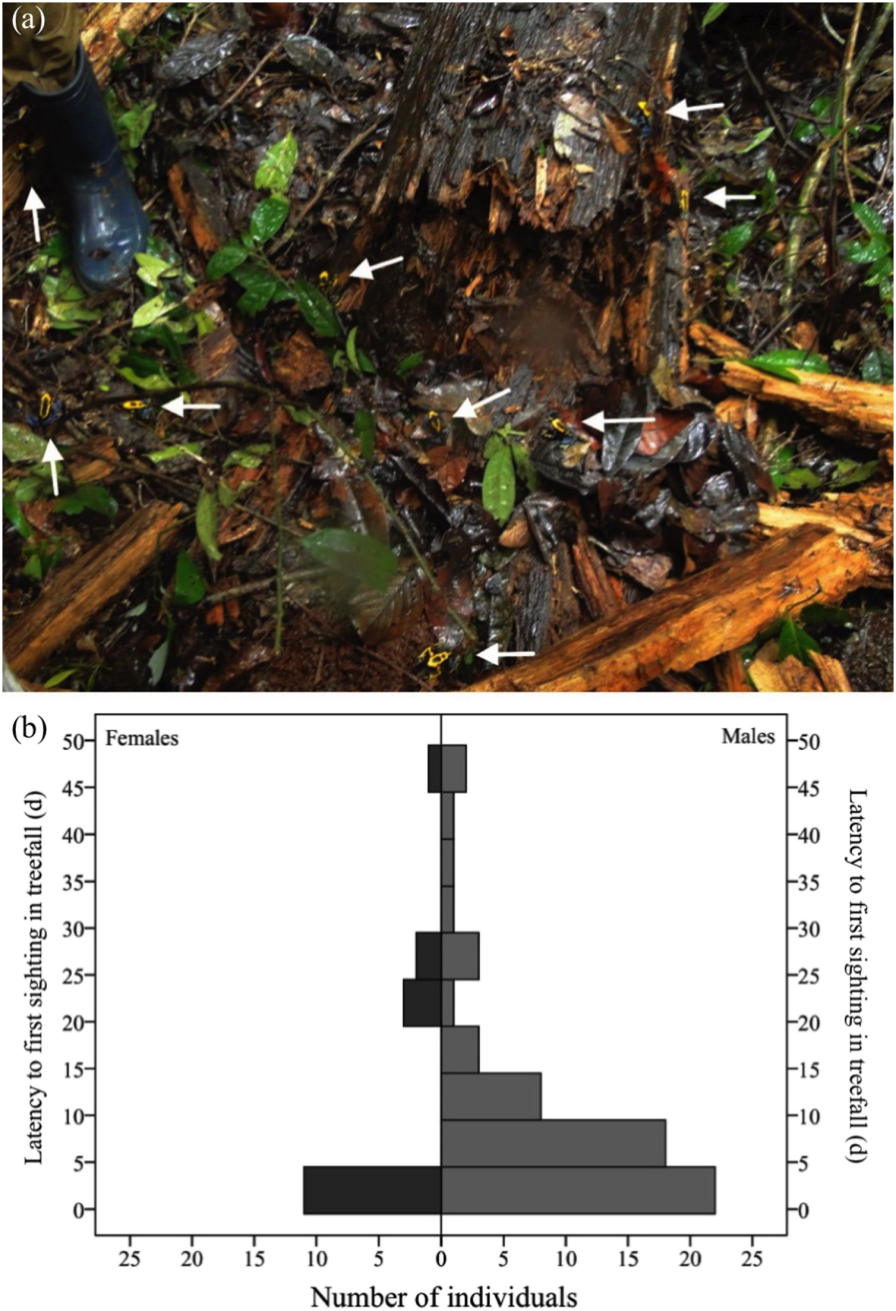
Dozens of adult *D. tinctorius* (pointed at by the arrows) can aggregate at once at a newly formed treefall gap (A). There are no sex differences in immediate arrival in a newly formed gap (χ^2^= 0.08 df = 1, *P* = 0.778), but males are more likely to be found in treefall gaps in the long term ((B) χ^2^ = 11.137, df = 1, *P* = 0.001). Photo credit: Bibiana Rojas.

A total of 113 individuals (55 females and 58 males) arrived in the nine fresh gaps studied either the same day or one day after their formation (Fig. 4A). Males were as likely as females to arrive within this timespan (χ^*2*^= 0.08, df = 1, *P* = 0.778). In the long term (i.e., up to 51 days after the occurrence of the treefall), however, more males than females were found in treefall gaps (χ^*2*^= 11.137, df = 1, *P* = 0.001). Only 77 (new) individuals were recorded after two days of treefall occurrence (i.e., after the addition of water bowls), 60 males and 17 females (Fig. 4B).

### Vocal behavior

**Figure 5:**
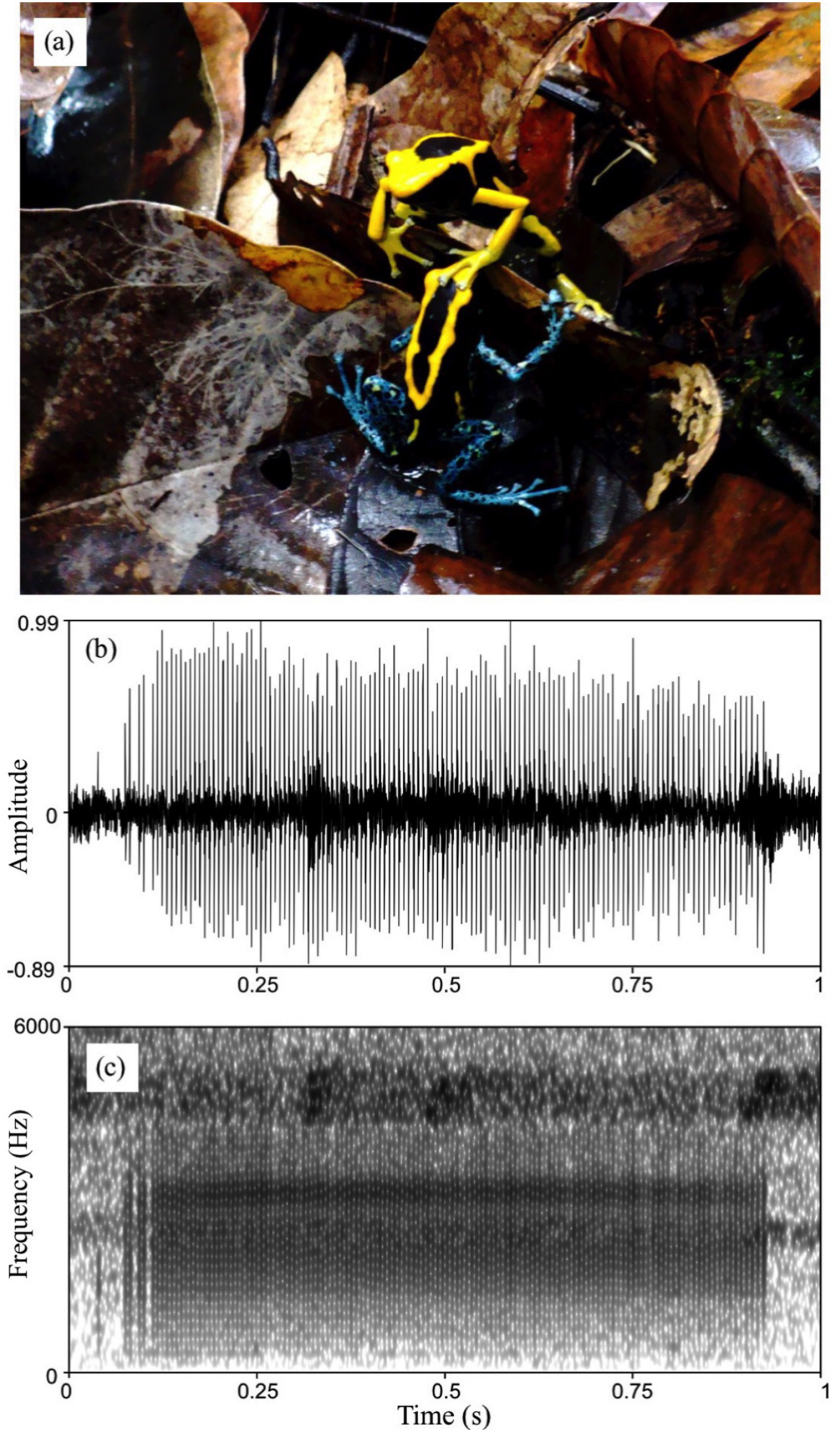
Courtship in *D. tinctorius*. (A) Example of tactile interactions observed between courting individuals: a female with a forelimb on a male’s head. (B) Waveform and (C) spectrogram of *D. tinctorius* call recorded from close range (approx. 30 cm) during courtship. The normalized waveform reveals the relative amplitude modulation and the pulsating structure of the call (pulse rate = 154 Hz); the spectrogram (FFT window length = 0.01 s, Gaussian window, frequency range 0–6,000 Hz) show the broadband spectral structure of the call with dominant frequency band centered around 3,150 Hz. Photo credit: Bibiana Rojas.

*Dendrobates tinctorius* produces a call that can be described as a very low intensity “buzz”, *sensu* Myers & Daly (1976). The call is audible to humans only from within a few meters; at times males inflate the vocal sac without anything audible to us from a distance of up to one m. Males call rarely and only when in courtship or during agonistic interactions with other males. We never observed a male calling alone. We were able to record and measure calls from two males in courtship and one in an agonistic interaction. Calls produced in courtship and agonistic contexts sounded similar to us and had similar acoustic parameters, although more recordings would be needed for a detailed comparison. All measured calls shared the same general structure: a short broadband burst of pulses produced at a high rate (Figs. 5B and 5C). The measured call duration was 0.55–0.98 s (mean = 0.76 s), the within-call pulse rate was 143–175 Hz (mean = 160 Hz), and the dominant frequency band centered around 2,700–3,270 Hz (mean = 3,109 Hz). For reference, a similar-sounding call was recorded from a different population of *D. tinctorius* in French Guiana (J. Sueur, 2019, personal communication) and is available online from the sound collection (sonothèque) of the National Museum of Natural History in Paris (https://sonotheque.mnhn.fr/sounds/MNHN/SO/2019-60).

### Courtship and egg laying

We found 47 pairs engaged in courtship (10 in 2009, 14 in 2010, and 23 in 2011), involving 40 males and 39 females. Courtship was observed throughout the day and lasted several hours, but we cannot be certain that we ever witnessed the beginning of a courtship session. In one case a courting pair was followed for nearly 7 h before oviposition took place. Courtship always consists of several bouts of moving together following each other (“pursuing”, sensu Silverstone (1973)) and stationary tactile interactions (Fig. 5A; Video S1) that are interrupted, for example, when one of the individuals starts to feed.

In general, each bout is initiated by tactile interactions in which the female repeatedly places one of her forelimbs on the male’s limbs, back or head, similar to what has been described for other species of poison frogs (Crump, 1972; Silverstone, 1973; Wells, 1978; Limerick, 1980). The male then faces her before moving away, followed by the female, in search of an egg-laying site. When a female stops following for several minutes, for example because she starts to forage, the male usually turns back and calls. Males also produce the same soft “buzz” calls during some tactile interactions and following bouts. On at least two instances we observed the male approaching the female and touching her head or back when she did not approach the male, as reported for *D. auratus* (Wells, 1978). Altogether, the courtship sequence in *D. tinctorius* appears to be very similar to that in *D. auratus* (Wells, 1978), with females taking the most active role. Both males and females vibrate the second digit of the hind legs at high frequency (“toe trembling”, sensu (Hödl & Amézquita, 2001); see Video S1) during courtship. Toe-trembling behavior can also be observed during foraging and agonistic interactions.

The courting pair does not seem to move over great linear distances (mean = 4.5 m; range, 0–8 m; *n* = 6), but moves in circles within an area of a few square meters instead. As courtship progresses, the pair stops at certain places under the leaves or inside a hollow trunk, and the female starts to move in circles on the same spot (“circling”, sensu Hödl & Amézquita, 2001) with alternating movements of her hind limbs in what appears as wiping of the leaves (see Video S2). The pair sometimes rests on the same spot for several minutes and the tactile interactions increase considerably during these breaks. The pair does this a few times, at different places (at least five in the case of the pair that we followed for about 7 h), before they choose the place where egg laying occurs, which appears to be selected by the female.

**Figure 6:**
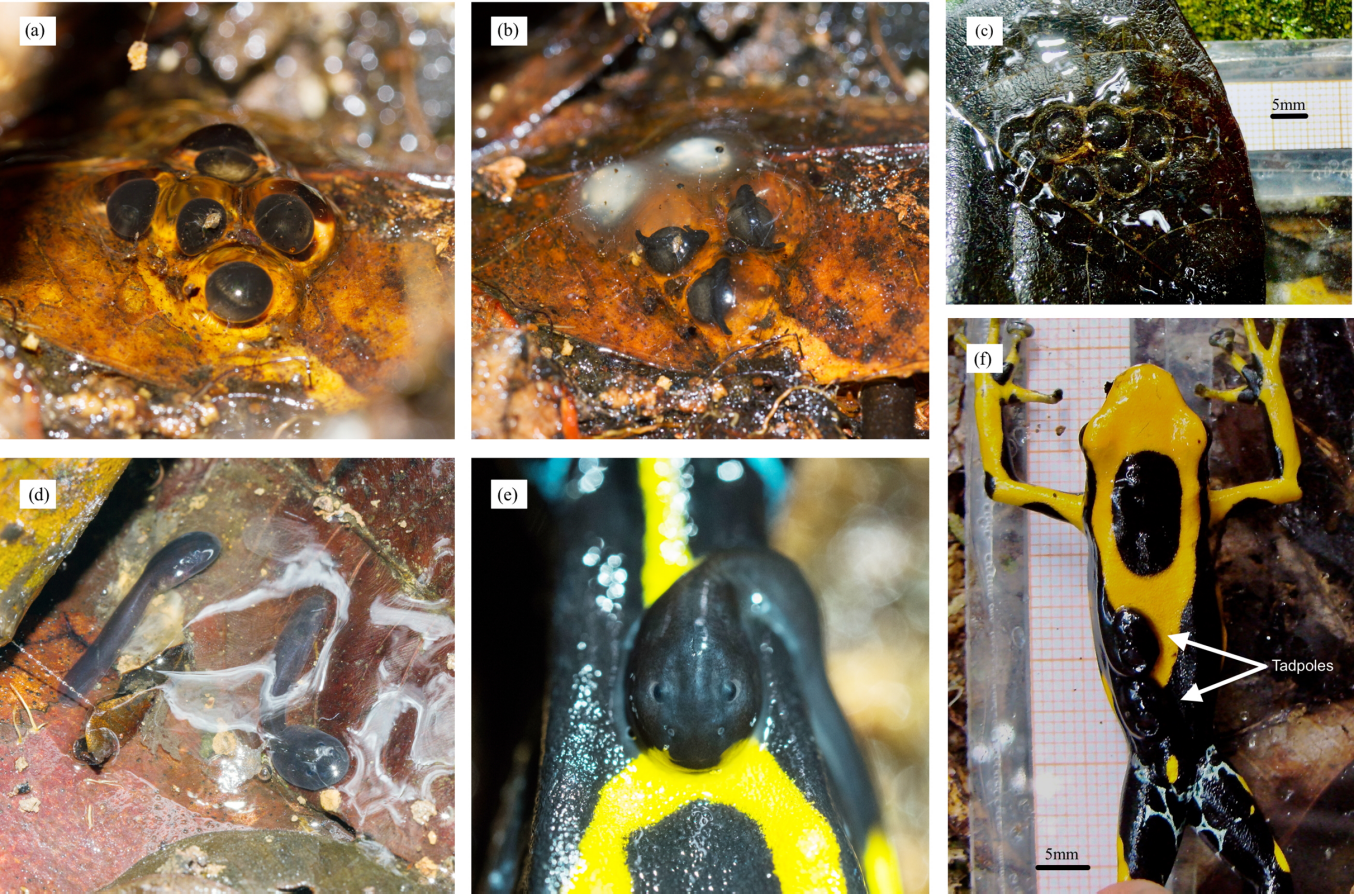
Clutch development in *D. tinctorius* in the wild. (A) Freshly laid clutch of five eggs; (B) The same clutch 5 days later. Note that two of the initial eggs have been infected by a fungus; (D) 15 days after egg laying, two surviving tadpoles are ready to be picked by the male and taken to a body of water where they will continue to develop until metamorphosis; (E) A tadpole attached to the male’s back. (C) A fresh clutch and (F) a male with two tadpoles on his back. (C) and (F) provide scales for size reference. Photo credits: Andrius Pašukonis (A, B, D, E) and Bibiana Rojas (C, F).

In addition to the clutches laid by pairs we followed during courtship (*n* = 3), which were laid under or inside small fallen logs on the ground, we found 18 clutches (for a total of 21) with 2–5 embryos (mean = 3.6) at different developmental stages. The eggs were laid under or within fallen logs and other wooden structures, leaf litter, palm bracts and leaves, and animal burrows, usually completely sheltered from the rain (Figs. 6A–6C; Video S2). Egg diameter is ~4.2 mm and hatching occurs after approximately 2 weeks (B. Rojas, 2011, personal observation; this study; Fig. 6A). Eleven clutches were followed during development and only 14 out of 46 embryos (30.4%) from eight out of 11 clutches survived until hatching. Other embryos did not develop, were destroyed by fungus, or disappeared. Twelve clutches in total were observed with 1–4 (mean = 2.2) tadpoles ready for male transport (Fig. 6C). Males were found occasionally sitting near or on top of egg clutches, most likely inspecting and moistening them, as has been reported for other poison frog species (Pröhl & Hödl, 1999; Wells, 1978; Weygoldt, 1980, 1987).

### Larval development and patterns of tadpole transport

**Figure 7:**
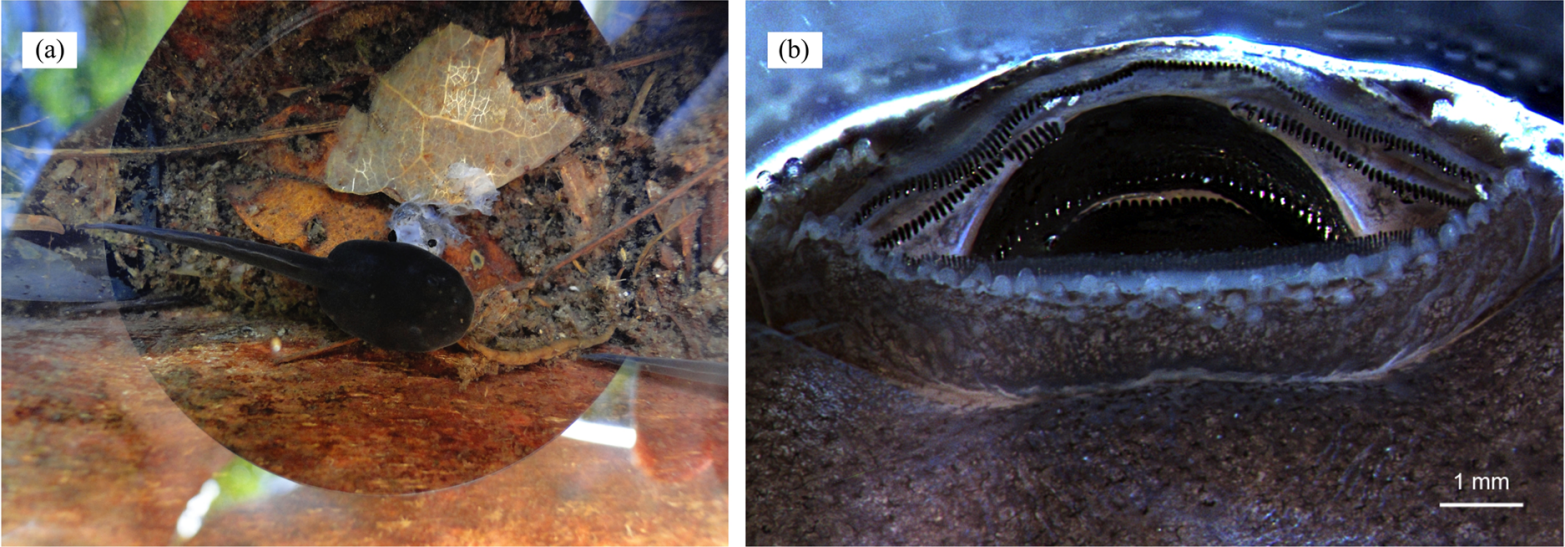
Cannibalism in tadpoles of *D. tinctorius*. (A) A cannibalistic tadpole with the remainings of its victim; (B) oral apparatus (anterior side up) of a stage 25 (Gosner, 1960) *D. tinctorius* tadpole. Photo credits: Bibiana Rojas (A) and Eva K. Fischer (B).

Hatching occurs after approximately 14 days (Fig. 6D), but the tadpoles may remain viable in the clutch for several days before being transported (A. Pašukonis, 2016, 2017, personal observation). The male eventually returns and sits on the clutch, allowing the tadpoles to wriggle on his back (Figs. 6D and 6E), and takes them to suitable bodies of water where they will remain unattended until metamorphosis, feeding on detritus and the larvae of some insects (e.g., Diptera and Odonata) and other frogs (Rojas, 2014), even conspecifics (Fig. 7A; Rojas, 2014, 2015). Tadpole mouthparts are well suited for their carnivorous diet, with hardened serrated jaw sheath (Silverstone, 1975; Fig. 7B). Size at metamorphosis ranges 10.94–15.62 mm (mean = 13.15 ± (SE) 0.24 mm, *n* = 24), and the color patterns are already completely visible in metamorphs (Fig. 2D).

**Figure 8:**
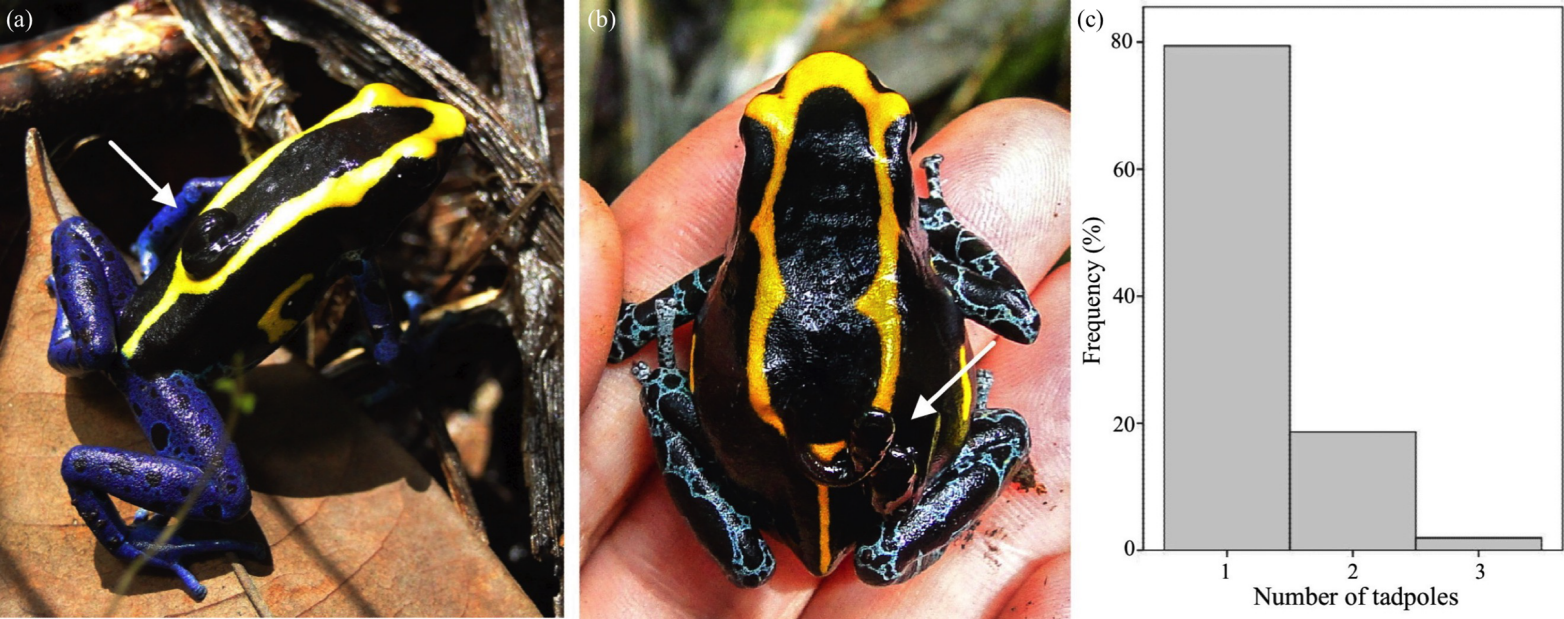
Tadpole transport in *D. tinctorius*. A male (A) and a (exceptional) female (B) with tadpoles on their back (indicated by white arrows). (C) Most individuals were found carrying one tadpole, but two and three tadpoles can also be carried at once. Photo credit: Bibiana Rojas.

We found 102 males (7 in 2009, 17 in 2010, and 78 in 2011) carrying one (Fig. 8A; 79.4%), two (18.6%), or three (2.0%) tadpoles (mean ± SE = 1.23 ± 0.05; Fig. 8C) ranging 4.78–6.87 mm long (from the tip of the snout to the base of the tail; mean ± SE = 5.52 ± 0.07 mm). On one exceptional occasion B. Rojas also found one female carrying two tadpoles with a visible difference in size (Fig. 8B). Pairs of tadpoles transported by a male simultaneously differed between 0.06 and 0.64 mm in size (mean ± SE = 0.25 ± 0.05). Some males carrying more than one tadpole were seen depositing one of them in a pool and leaving with the second tadpole still attached to their back, whereas other males were seen depositing their two tadpoles in the same pool, at the same time. Some males were also seen visiting more than one pool before the tadpole(s) detached from their back. The visits consisted of jumping into the pool and sometimes repeatedly diving inside for several minutes while the tadpole remained attached (see Video S1)

### Aggressive behavior

We observed 23 agonistic encounters involving both male–male (*n* = 10) and female–female (*n* = 13) pairs. On one occasion, a male shortly attacked a female while attacking another male but resumed courting the same female shortly after. The agonistic interactions ranged from short instances of chasing without any physical contact to prolonged continuous physical combat lasting at least 20 min.

**Figure 9:**
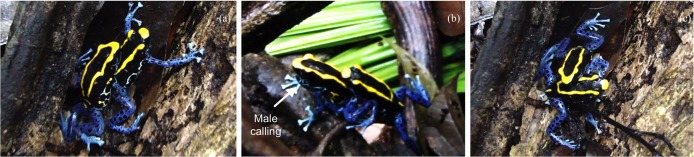
Images of an agonistic encounter between two male *D. tinctorius*. Physical combat involves pressing the opponent against the substrate (with either the forelimbs or the whole body) (A), wrestling (C), and kicking. Occasionally, males also vocalize during fighting, as seen by the inflated vocal sac in the (B). Photo credit: Bibiana Rojas.

In both sexes, the physical fights involved kicking, jumping on each other’s back, and pressing either the head or the dorsum against the substrate (Fig. 9; Video S1). In most cases, we were unable to identify the origin of the conflict, but it seemed to occur both in the presence (at least six times in our records) and absence of an individual of the opposite sex. While this was not always the case, both male and female aggressive interactions were observed while one of the contestants was involved in courtship (*n* = 3). For example, on one occasion, while observing a courting pair in which the female was following the male closely, a second female who had been under a log suddenly appeared and immediately assaulted the courting female. The intruding female jumped on top of the courting female, trying to press the body of the latter against the substrate. The courting female recovered, and tried to go on top of the intruder, and these alternating attacks rapidly became a seemingly intense physical combat, in which movements and attacks occurred at a high speed. The male turned away from the females and started to call at a high repetition rate. The combat lasted for about ten minutes at the end of which the intruder female moved away, presumably defeated by the courting female. The courting pair continued to be together for a couple more hours until egg laying occurred. On other occasions, we noticed the presence of a female in the vicinity of two males engaged in a physical combat after which one of the males courted the female while the other moved away. Some agonistic interactions both between males and between females occurred with no visible involvement of the opposite sex. Interestingly, on two occasions males carrying tadpoles were also seen engaged in physical combats with other males.

## Discussion

The purpose of this study was to provide basic information about various aspects of the natural history of *D. tinctorius* in the wild that could be used as background knowledge for future research on the behavioral ecology, evolution and conservation of the species. We describe their reproductive and social behaviors, habitat use, and their remarkable colonization of tree-fall gaps as soon as they occur. The implications of these findings, as well as some hypotheses derived from our observations, are discussed below.

*Dendrobates tinctorius* males were most often found climbing, foraging, and hiding around forest structures, such as dead logs, fallen branches, roots, tree buttresses, and palm bracts. These structures are used as oviposition sites (this study), and are also the types of structures that accumulate rainwater, forming pools where newly hatched tadpoles are deposited (Rojas, 2014). Females, in contrast, were more often found foraging on the ground in open areas. Sex differences in microhabitat use might thus be related to differences in parental duties, as males periodically attend developing clutches and are in charge of tadpole transport and deposition. Forest structures can also be used by both sexes as communal retreats during dry periods (Born et al., 2010), as has also been reported for *D. truncatus*, a closely related species (Gualdrón-Duarte et al., 2016). Such microhabitat likely provides higher humidity and shelter from potential predators. Differences between the sexes in habitat use could also reflect differences in feeding rates and foraging activity, or differences in patterns of space use. These aspects are known to be different, for example, between male and female *Oophaga pumilio* (Donnelly, 1989a, 1989b, 1991).

Both males and females invade tree-fall gaps within 3 days of their formation, possibly attracted by the sudden abundance and diversity of food (B. Rojas, 2011, personal observation). In fact, frogs captured in recently formed tree-fall gaps have shown a tendency to have more prey items in their stomach than frogs caught in the closed forest (Born et al., 2010). The mechanism by which these frogs detect and locate treefalls remains unidentified. However, the sound and seismic cues produced during a treefall might be sufficient, as some frogs are known to detect vibrational signals from conspecifics (Caldwell et al., 2010; Lewis & Narins, 1985), heterospecifics (Warkentin et al., 2007) and rain (Caldwell, McDaniel & Warkentin, 2010); these three kinds of signals are presumably much weaker than those produced by a treefall. Low-frequency seismic cues could be detected at long distances but are short in duration. Thus, it is possible that strong olfactory cues and light gradients produced by a fresh treefall provide the additional information needed for orientation.

Males seem to stay longer and keep arriving in this newly created habitat at later stages than females. Several factors may be influencing this difference between the sexes. A resource supplementation study that was conducted in parallel at the same treefall sites found higher tadpole-deposition rates in artificial pools placed at recent tree-fall gaps in comparison to pools in the closed forest (Rojas, 2015). These findings suggest that the availability of new places for tadpole deposition is one of the drivers of tree-fall gap invasion in this species. Unfortunately, we cannot disentangle the sex differences due to resource supplementation (i.e. addition of bowls with water, see Rojas, 2015) from naturally occurring differences. However, natural pools frequently form in trunks of freshly fallen trees (B. Rojas and A. Pašukonis, 2011, 2013, 2016, 2019, personal observations; Video S1) and the addition of artificial pools should only quantitatively, but not qualitatively, change the value of this habitat. In fact, tadpole-carrying males can be seen at a new treefall gap even on the day of its formation (Rojas, 2015). Moreover, as suggested by Born et al. (2010), the simultaneous presence of many individuals (Fig. 4A) can make tree-fall gaps a perfect mating arena. At least one other neotropical rainforest frog species, albeit with a different life history, is known to aggregate at young canopy gaps and form choruses to attract females (*Agalychnis (=Cruziohyla) calcarifer*; Marquis, Donnelly & Guyer, 1986). If *D. tinctorius* mated in the treefall areas, then males would need to stay in the area to attend the clutches and transport the tadpoles, while the females could return to their previous home areas. Further studies on the sex differences in microhabitat and space use, and how these are influenced by the mating systems and parental roles, are needed to better understand this aspect of *D. tinctorius*’ life history.

One of the most unusual aspects of *D. tinctorius*’ reproductive behavior, and likely one of the reasons why their behavior has only recently started to be studied in the wild, is the lack of advertisement calls. Most male frogs, including other dendrobatids, use calls to attract females and to repel rival males (Erdtmann & Amézquita, 2009; Gerhardt & Huber, 2002; Santos et al., 2014), making them also easier to locate by researchers. The structure of these calls shows great variation across the poison frog family (Myers & Daly, 1976; Erdtmann & Amézquita, 2009), and a recent large-scale comparative study (Santos et al., 2014) argued that a reduced predation pressure has facilitated this diversification in acoustic signals in aposematic species. Paradoxically, and in contrast to the vast majority of frogs, aposematic *D. tinctorius* appears to have lost the advertisement function of its call altogether. Two closely related species, *D. auratus* and *D. truncatus*, also vocalize less frequently and at lower intensities than most other poison frogs, but still use calling both for territorial advertisement and courtship (Wells, 1978; Summers, 1989; Erdtmann & Amézquita, 2009; Gualdrón-Duarte et al., 2016). What factors drove or facilitated the loss of typical calling behavior in *D. tinctorius* remains an intriguing evolutionary puzzle. Despite their toxicity, recent studies indicate that predation risk by naïve predators may still be an important selective pressure (Noonan & Comeault, 2009; Comeault & Noonan, 2011; Rojas, Rautiala & Mappes, 2014), suggesting that the increased exposure associated with prominent calling behavior should be selected against. However, this situation is not exclusive to *D. tinctorius*, and poison frogs in the genus *Oophaga*, for instance, have kept their advertisement calls and an active vocal behavior despite their conspicuous coloration (Pröhl, 2003; Vargas-Salinas & Amézquita, 2013; Willink et al., 2013). On the other hand, male and female *D. tinctorius* tend to segregate in and around tree-fall gaps and other forest structures, potentially facilitating mating pair formation by direct encounter without the need of acoustic signals. We speculate that such microhabitat segregation and the availability of putative visual signals for communication in a diurnal colorful frog (discussed below) has promoted the loss of the advertisement call in *D. tinctorius*.

Male *D. tinctorius* use calls, however, in courtship and agonistic interactions. The courtship call resembles a lower intensity version of calls produced by closely related species, such as *D. auratus* and *D. truncatus* (Wells, 1978, Gualdrón-Duarte et al., 2016; B. Rojas, 2011, personal observation). In addition to advertisement calls, many other dendrobatid frogs use soft courtship calls (e.g., Roithmair, 1994), which are to facilitate the contact with the female during the prolonged courtship while reducing the potential detection and conflict with competitor males (Wells, 2007). Courtship calls may also stimulate the ovulation in females, signal territory ownership or function as visual signals because of the slow and prominent vocal sac inflation. In *D. tinctorius*, males often take a distinct elevated posture when calling both during courtship and agonistic encounters, and this posture is retained at times in the absence of vocalizations. This so-called “upright posture” is thought to function as a visual signal in both contexts (Hödl & Amézquita, 2001).

Visual signals (de Luna, Hödl & Amézquita, 2010; Narins, Hödl & Grabul, 2003; Santos et al., 2014; Summers et al., 1999) and tactile interactions (Bourne et al., 2001; Pröhl & Hödl, 1999; Summers, 1992) have long been thought to play an important role in poison frog communication. Aspects of dorsal coloration, for example, are known to influence mating decisions (Summers et al., 1999; Maan & Cummings, 2008) and agonistic encounters (Crothers, Gering & Cummings, 2011) in at least one species of poison frog, *O. pumilio*. However, in *O. pumilio* and other species, acoustic signals still mediate the initial mate attraction (Dreher & Pröhl, 2014; Lötters, Reichle & Jungfer, 2003; Pröhl, 2003) and male–male competition (Amézquita et al., 2006; Bee, 2003; Crump, 1972; Ringler et al., 2011; Rojas, Amézquita & Delgadillo, 2006; Tumulty et al., 2018).

In the absence of advertisement calls, the use of tactile stimuli and both static (such as dorsal color patterns) and dynamic visual signals most likely plays a predominant role in *D. tinctorius* communication. Dorsal color patterns might mediate mate choice (Rojas, 2017), given that individuals follow each other for a considerable amount of time while searching for a suitable place for oviposition. Males have been found to have a higher proportion of yellow in their dorsal area than females in our study population (Rojas & Endler, 2013). This has been suggested to be particularly beneficial during tadpole transport (Rojas & Endler, 2013), a task that requires long displacements and prolonged exposure (Pašukonis, Loretto & Rojas, 2019), especially when climbing trees. Male coloration might thus indicate parental male quality and be subject to sexual selection (Rojas, 2017). The variable coloration patterns on these frogs’ front, forelimbs, and flanks, could also have the potential to be used as signals, as a lot of the time the frogs are either facing or next to each other during courtship (Rojas, 2012). These color patterns may be used for species, sex, or even individual recognition from the distance. Individual recognition has not been shown in any amphibian, but the relatively complex social behavior, the lack of acoustic communication, and the repeated encounters in their shared microhabitat may have promoted such ability in *D. tinctorius*.

Both male and female *D. tinctorius* engage in intra-sex aggression that may escalate to intense physical combats, which involve chasing, wrestling, and prolonged pressure over the opponent’s head or dorsum. These types of behaviors have been also reported for the closely related *D. auratus* (Summers, 1989; Wells, 1978) and *D. leucomelas* (Summers, 1992). Aggression in male poison frogs is usually a result of male competition for mates and territorial defense mediated by acoustic interactions (reviewed in Pröhl, 2005). To the best of our knowledge, males of all dendrobatid species studied to date show some degree of territoriality (Pröhl, 2005). *Dendrobates tinctorius* seems also unusual in this respect, as they do not appear to defend exclusive areas. Similar to Born et al. (2010), we have observed males foraging in close proximity without aggressive escalations in large aggregations around fresh tree-fall gaps, as well as around structures where a few males might take refuge. We observed that the presence of individuals of the opposite sex, especially during courtship, was the cause of some of the agonistic encounters both between males and between females. Inter-female aggression has been also reported for *Mannophryne trinitatis* (Wells, 1980), *D. auratus* (Wells, 1978; Summers, 1989), *D. leucomelas* (Summers, 1992), and *O. pumilio* (Meuche, Linsenmair & Pröhl, 2011). Just like in *D. tinctorius*, in the closely related *D. auratus*, tadpoles are cannibalistic and males may deposit tadpoles from multiple clutches in the same pool (Summers, 1989, 1990). As suggested for *D. auratus*, female aggression thus might be the result of attempts to monopolize males and reduce the potential competition and risk of cannibalism by unrelated tadpoles in shared pools.

Interestingly, we also observed aggressive interactions that seemingly did not involve a third individual, suggesting aggression triggers other than access to mates. These observations should, however, be interpreted with caution, as we cannot be certain that a third individual was not hiding in the area. In addition to mating context, aggression in some dendrobatid frogs has been linked to defense of shelter and feeding areas (Wells, 1980; Meuche, Linsenmair & Pröhl, 2011), but *D. tinctorius* does not appear to defend exclusive territories (Born et al., 2010). Some of the aggressive interactions resulted in the defeated individual being chased away, as if in a territorial displacement, but others terminated with both individuals continuing to forage nearby. This hints at an establishment of dominance hierarchies between opponents, which we suggest could be the result of repeated encounters of individuals in their shared microhabitat. Dominance hierarchies are well documented in all other vertebrates, where dominant individuals get preferential access to food, mates, and shelter (reviewed in Huntingford & Turner, 1987). However, the formation of such potential hierarchies has not been described for any anuran species in the wild, despite being suggested to arise among poison frogs in captivity (Zimmermann & Zimmermann, 1988), where they mediate conflict resolution at least in *O. lehmanni* (Rojas, 2002). This is, therefore, a subject that merits further investigation.

Aggressive behavior and territoriality in *D. tinctorius* might be context-dependent and related to population density, variation in food abundance and other resources, such as structures for shelter or oviposition. In the absence of vocalizations, *D. tinctorius* may be using visual signals to get information about the fighting abilities of their opponents, as it has been reported for male *O. pumilio* (Crothers, Gering & Cummings, 2011; Crothers & Cummings, 2015), and settle their conflicts before escalating to physical combats (Rojas, 2017). Social behavior in *D. tinctorius* is a promising avenue of research, which could provide insights into the evolution of visual communication and factors influencing anuran aggressive and territorial behavior in the absence of acoustic communication.

Egg clutches at our study site have high mortality and are much smaller than those reported in captivity, which may have up to 14 eggs (Lötters et al., 2007). This does not seem to be an exception, as levels of hatching failure of up to 80% have been previously reported for *O. pumilio* (Pröhl & Hödl, 1999). Loss of most eggs or embryos is likely due to predation (Juncá & Rodrigues, 2006), or to fungal infections (Fig. 6B). On one occasion, we observed a female unrelated to the clutch on top of the missing eggs, indicating possible cannibalism. This behavior has been previously reported in *D. auratus* as a mechanism of intra-female competition (Summers, 1989, 1990). Males of *O. pumilio* are also known to be able to cannibalize the eggs of rival males (Weygoldt, 1980).

Upon hatching, males take tadpoles, either all of them or one at a time, to bodies of water. The latter is thought to be the case of most *Dendrobates*, although the evidence supporting this pattern comes mostly from observations in captivity (Lötters et al., 2007). Transport of single tadpoles, one by one, implies several trips between the place where clutch was laid and the pools, a task that has been shown to require remarkable spatial abilities (McVey et al., 1981; Pašukonis, Loretto & Hödl, 2018; Pašukonis et al., 2013, 2016; Pichler et al., 2017; Stynoski, 2009; Pašukonis, Loretto & Rojas, 2019) and probably a high energetic cost (see discussion in Beck et al. (2017) and Summers (2019)). In *D. tinctorius*, males carry one or two (sometimes three) tadpoles at a time. In combination with the high clutch mortality rates observed, this suggests that males often take all the larvae that survive within a given clutch at once. However, we have observed at least a few instances in which males take one tadpole to a pool and then return to get the rest (Pašukonis, Loretto & Rojas, 2019). Tadpoles transported at the same time on a male’s back may differ visibly in size. Whether these size differences reflect within-clutch size variation or the transport of tadpoles of different clutches requires further investigation. In *D. auratus*, males have been seen moistening a fresh clutch and a hatching clutch within the same hour in captivity (Wells, 1978), and attending multiple clutches of different stages in the field (Summers, 1990; Wells, 1978). This is likely to be the case in *D. tinctorius* as well. Size difference between tadpoles transported simultaneously was particularly noticeable in the tadpoles on the back of the only female found performing these duties. While rare (1 in >100 tadpole transport events reported here), it seems that tadpole transport might be taken over by females if males go missing, as reported in other species of poison frogs (Myers & Daly, 1983; Tumulty, Morales & Summers, 2014) and experimentally demonstrated in *Allobates femoralis* (Ringler et al., 2015).

We observed tadpole deposition in different water-holding structures in the forest, from palm bracts on the ground to tree holes high up. However, the specific characteristics that influence pool choice by a male and favor successful tadpole development are currently unknown. It has been previously suggested that, despite the high levels of tadpole cannibalism, parents might use the presence of larger tadpoles as a cue of pool quality. Whether existent tadpoles in the pools chosen by males are related to the new tadpole is a task for future research, likely using the microsatellite markers developed for *D. tinctorius* (Ringler et al., 2012). Regardless, the presence of large tadpoles may indicate that basic requirements, such as sufficient nutrients and water stability, have been met to allow tadpole development (Rojas, 2014). Even less is understood about the role that *D. tinctorius* plays in the ecology of other phytotelm-breeding anurans, especially considering that most species are restricted to terrestrial or arboreal habitats; meanwhile, *D. tinctorius* and their carnivorous tadpoles are capable of exploiting pools at all heights (Gaucher, 2002). How *D. tinctorius* finds canopy pools is unknown, but it has been speculated that they may eavesdrop on the calls of treefrog species such as *Trachycephalus resinifictrix* and *T. hadroceps*, which breed in arboreal water bodies (Gaucher, 2002). We further hypothesize that enlarged male toe-pads (apt for climbing) and aposematic coloration (Rojas & Endler, 2013) gave *D. tinctorius* access to a wider variety of aquatic habitats despite being exposed to would-be predators for prolonged periods of time during tadpole transport.

Approximately 43% of the amphibian species worldwide are experiencing population declines (Stuart et al., 2004), largely as a consequence of the spread of a deadly disease caused by the fungus *Batrachochytrium dendrobatidis* (*Bd*) (Bower et al., 2017; Lips, 2016; Lips et al., 2006; Scheele et al., 2019). Despite having a low *Bd* prevalence compared to species in other families and regions (e.g., Flechas, Sarmiento & Amézquita, 2012), phytotelm-breeding dendrobatids, including *D. tinctorius*, have been found to have the highest prevalence of *Bd* in recent studies done in French Guiana (Courtois et al., 2015). While *Bd* research has been mostly focused on adult frogs, it is known that tadpoles can also get infected due to their keratinized mouthparts (Berger et al., 1998; Blaustein et al., 2005). Most importantly, tadpoles, regardless of whether or not they express the disease, can be important vectors of *Bd* to adults. However, there is currently no information on *Bd* prevalence in *D. tinctorius* tadpoles, or studies assessing the presence of *Bd* in the pools where tadpoles develop, despite reports of *Bd* occurrence in phytotelmata and phytotelm-breeders in other Neotropical areas (Cossel & Lindquist, 2009; McCracken et al., 2009). Furthermore, the dispersal of *Bd* outside large bodies of water may imply an amphibian vector (Kolby et al., 2015), and movement patterns are known to directly affect the dynamics of disease spread (Daversa et al., 2017). Thus, we urge studies evaluating the role of *D. tinctorius* adults as *Bd* vectors both across different pools at the ground level and across forest strata (i.e., from the forest floor to the canopy).

Another major threat for anurans is habitat destruction (Cushman, 2006). While our study population occurs within in a natural reserve, many populations of *D. tinctorius* are in unprotected areas, which are under threat primarily by gold mining-driven deforestation. It is estimated that approximately 41% (~684 km^2^) of the deforestation in the South American tropical rainforest between 2001 and 2013 occurred in the so-called Guianan moist forest ecoregion due to gold mining activities (Alvarez-Berríos & Aide, 2015). Because *D. tinctorius* is often distributed in small patchy populations (Noonan & Gaucher, 2006), deforestation even at the small scales used for gold mining, can have a detrimental, probably irreversible effect on the life histories and survival of this species and other phytotelm-breeders. We thus fully support the long-term monitoring strategies suggested by Courtois et al. (2015) and currently implemented across several nature reserves in French Guiana (e.g., http://www.reserve-tresor.fr/en/our-actions/studies-and-surveys/herpetology) to allow the timely assessment of changes in population size and sudden declines, especially of “sentinel species” such as *D. tinctorius* (Courtois et al., 2013, 2015). Likewise, we endorse the recent initiative of declaring *D. tinctorius* a protected species in French Guiana. These types of strategies, together with basic research on the natural history of threatened species, are key not only for the formulation of successful conservation policies, but also for the education and future engagement of public essential for the preservation of wildlife at a local scale.

## Conclusions

While natural history is unarguably the basis of scientific progress, natural history studies currently tend to be undervalued and are thus in decline. Yet, there have been recent attempts to reinstate the relevance of natural history, emphasize its role in scientific breakthrough and revive our interest in it (Hampton & Wheeler, 2012; Anderson, 2017). Neotropical poison frogs are a great example of how detailed observations of natural history in the wild can lead, and have led, to revolutionary hypothesis-driven studies that have changed several paradigms about amphibian behavior and ecology. The dyeing poison frog (*D. tinctorius*), however, remained understudied for a long time possibly due to the marked absence of a regular calling behavior, which is a trademark among species of poison frogs and their close relatives. By compiling our multi-year observations of a wild population of *D. tinctorius* in French Guiana, we aimed to provide a solid basis for future fundamental and applied research on different aspects of the ecology, behavior, and conservation of this species, in particular, and of poison frogs, in general. We (1) found striking differences in habitat use, so that males are more often associated with complex structures whereas females tend to favor open areas; (2) document the remarkable invasion of tree-fall gaps within one or two days of their occurrence; (3) describe their call, as well as their courtship and parental care behaviors; (4) report the occurrence of aggressive behavior in both sexes; and (5) discuss how the knowledge generated by this study could set the grounds for further research on spatial ecology, conflict resolution, parental care, sexual selection, disease transmission, and long-term population monitoring in this and other species of poison frogs.

## Supplemental Information

10.7717/peerj.7648/supp-1 Supplemental Information 1 The dyeing poison frog *Dendrobates tinctorius*: fighter, lover, cannibal, and parent.

10.7717/peerj.7648/supp-2 Supplemental Information 2 Egg laying in *Dendrobates tinctorius* in the wild.

10.7717/peerj.7648/supp-3 Supplemental Information 3 Habitat by sex. Each row is an individual for whom the type of habitat upon sighting/capture was recorded in 2010. Columns correspond to the individual ID, sex, and type of habitat in which it was found (0 = open areas/leaf litter; 1 = associated with structures such as logs, roots, buttresses, etc.)

10.7717/peerj.7648/supp-4 Supplemental Information 4 Dtinctorius Size At Metamorphosis. Size at metamorphosis in mm for each individual.

10.7717/peerj.7648/supp-5 Supplemental Information 5 Latency to arrival in treefall. Each row is an individual that arrived in a treefall. The columns correspond to the individual ID, sex and latency to arrival in a treefall.

10.7717/peerj.7648/supp-6 Supplemental Information 6 Size of transported tadpoles in *D. tinctorius*.

## Additional Information and Declarations

### Field Study Permissions

The following information was supplied relating to field study approvals (i.e., approving body and any reference numbers): Our research was approved and authorized by the scientific committee of the Nouragues Ecological Research Station.

### Data Availability

The following information was supplied regarding data availability: The raw data are available as Supplemental Files, at the University of Jyvaskyla, and figshare: Rojas, B. & Pašukonis, A. 2019: Original data for article: From habitat use to social behavior: natural history of a voiceless poison frog, *Dendrobates tinctorius*—JYX, University of Jyväskylä. DOI 10.17011/jyx/dataset/65265. Rojas, Bibiana; Pašukonis, Andrius (2019): From habitat use to social behavior: natural history of a voiceless poison frog, *Dendrobates tinctorius*—Supplementary Videos. figshare. Media. DOI 10.6084/m9.figshare.9641822.v2.
